# Seedling Stage Strategies as a Means of Habitat Specialization in Herbaceous Plants

**DOI:** 10.1371/journal.pone.0023006

**Published:** 2011-07-29

**Authors:** Dirk-Jan ten Brink, Hans Henrik Bruun

**Affiliations:** 1 Biodiversity Group, Department of Biology, Lund University, Lund, Sweden; 2 Center for Macroecology, Evolution and Climate, Department of Biology, University of Copenhagen, Copenhagen, Denmark; Purdue University, United States of America

## Abstract

The regeneration niche has been little investigated in studies of community assembly and plant distribution. We examined adaptive associations between seedling traits and habitat specialization. Two habitat contrasts were investigated across several evolutionary lineages of angiosperms: species specialized to forest vs. open habitats and to dry vs. wet habitats. We also tested whether effects of shade and drought vary independently or, alternatively, if shade may amplify effects on drought-stressed plants. Seedling response in terms of growth rate, height, slenderness, specific leaf area (SLA) and degree of elongation (longest internode; petiole or leaf-sheath depending on species' morphology) to light and watering treatments was assessed. We used a factorial design involving three light regimes and two watering frequencies. The open-shaded habitat contrast and the dry-wet habitat contrast were investigated using six and five pairs of congeneric species, respectively. The congeneric species pair design controlled for confounding effects of evolutionary history prior to divergence in habitat specialization. Seedling growth rate generally decreased with shade and reduced watering frequency. Plant height was generally largest at intermediate light. Specialization to shaded habitats was associated with a more conservative growth strategy, i.e. showing a more modest growth response to increasing light. Species from all habitats showed the highest relative elongation at intermediate light, except for the moist-habitat species, for which elongation increased with shade. Contrary to our expectations, species from dry habitats grew bigger than species from moist habitats in all treatments. SLA responded to the light treatment, but not to watering regime. The contrasting light and moisture conditions across habitats appear to not have selected for differences in SLA. We conclude that seedling phase strategies of resource allocation in temperate herbs contribute to their habitat specialization. Habitat-specific seedling strategies and trade-offs in response to resource availability and environmental conditions may be important to adaptive specialization.

## Introduction

The assembly of plant communities may be seen as a selection process by which species from the species pool are sorted through abiotic and biotic filters [1], [2]. Filtering acts upon plant traits and either allows or denies species' establishment in habitats. This is predicted to lead to trait convergence at the between-habitat scale as a result of the general abiotic regime, whereas diversifying trait filters may operate at the within-community scale [3]. At the between-habitat level, functional plants traits can be said to correspond to the beta-niche [4], [5]. Within habitats, plant interactions, e.g. resource competition, and others processes, are thought to determine local species coexistence based on alpha-niches [4], [5], [6]. Filtering takes places at all plant life cycle stages, but the importance to habitat specialization of traits and requirements at the regenerative stage have been little investigate in studies of community assembly and plant distribution. Aspects of the regeneration niche [7], like seed germination, seedling establishment and early seedling survival, must be of primary significance to the establishment and long-term survival of populations. Germination cueing has previously been shown to be important in habitat specialization of temperate forest herbs [8], [9].

In this paper, we test the association between seedling traits and habitat specialization across several evolutionary lineages by using congeneric species pairs from contrasting habitats. Our focus is on herbaceous vascular plant species specialized to two broad sets of contrasted habitat conditions, shaded vs. open habitats and dry vs. moist habitats. The congeneric species pair selection ensures phylogenetic independence because the pairs are independent replicates of evolutionary divergence in habitat specialization. Furthermore, potential confounding effects of unmeasured traits due to shared evolutionary history can be excluded [10], [11].

Another objective of the study was to test for the combined effects of water and light availability, the two major axes of variation among the habitats in our study, in relation to the adaptation of species to contrasting habitats. Some theoretical studies resulted in contrasting hypotheses; Smith and Huston [12] predicted an amplified effect of drought on shaded plants, driven by a trade-off in shade and drought tolerance, whereas Holmgren et al. [13] hypothesized the effect of drought to be strongest at high and low light levels and to be weaker in intermediate shade. Empirical studies have found shade to alleviate drought effects or drought to have proportional effects across irradiance levels [14], [15], [16], [17]. Morphological, phytochrome-mediated shade avoidance responses include elongation of leaves, petioles and/or internodes [18], resulting in more slender plants, i.e. having an increased height to biomass ratio. Shade tolerance in plants is, among other things, characterized by an increased leaf area per unit leaf mass (specific leaf area, SLA) [19]. Leaf area is also influenced by water availability, and drought stressed plant often develop leaves with a reduced SLA [20]. A lower SLA reduces the potential growth rate, thereby reducing biomass accumulation in plants [19].

To test the adaptive habitat specialization of seedlings and their response to combined shade and drought stress, we performed a greenhouse experiment, in which we varied light availability by manipulating photosynthetically active radiation (PAR) and red to far-red ratio (R:FR) as well as watering frequency. Besides testing the before-mentioned hypotheses on the combined effects of drought and shade, we address the following hypotheses: Shade-adapted plants, as compared to open-habitat plants, 1) are less affected in growth rate with decreasing light, 2) exhibit a weaker shade avoidance response and 3) have a greater SLA across all light levels. We also predict seedling mortality to increase with decreasing light and decreasing watering frequency and to be lower among shaded-habitat species than for open-habitat species and lower among dry-habitat species than for moist-habitat species. Finally, we hypothesize specialization to dry habitats to be associated with a smaller growth reduction in response to drought than species from moist habitats due to higher water use efficiency and lower SLA; and we hypothesize growth rate to be lower for plant species from dry habitats than for species from moist habitats.

## Methods

### Species and seed selection

We selected 18 herbaceous species to form 11 congeneric species pairs with contrasting habitat preference (Table 1). The shaded-open habitat contrast was represented by six species pairs and the dry-moist habitat contrast by five species pairs. Two single species and one species pair were used in both contrasts. For plant nomenclature, we followed Flora Europaea [21].

**Table 1:** Congeneric species pairs used in the study and their respective plant traits analysed for their elongation response.

Family	Open-habitat species	Elongation measure	Shaded-habitat species	Elongation measure
*Poaceae*	*Bromus hordeaceus*	internode	*Bromus benekenii*	internode
*Cyperaceae*	*Carex ovalis*	leaf-sheath	*Carex sylvatica*	leaf-sheath
*Poaceae*	*Festuca arundinacea*	leaf-sheath	*Festuca gigantea*	leaf-sheath
*Rosaceae*	*Geum rivale*	petiole	*Geum urbanum*	petiole
*Polygonaceae*	*Rumex crispus*	petiole	*Rumex sanguineus*	petiole
*Caryophyllaceae*	*Silene latifolia*	petiole	*Silene dioica*	petiole

	Dry-habitat species	Elongation measure	Moist-habitat species	Elongation measure
*Asteraceae*	*Achillea millefolium*	petiole	*Achillea ptarmica*	internode
*Poaceae*	*Agrostis capillaris*	internode	*Agrostis stolonifera*	internode
*Cyperaceae*	*Carex ovalis*	leaf-sheath	*Carex lepidocarpa*	leaf-sheath
*Rosaceae*	*Geum urbanum*	petiole	*Geum rivale*	petiole
*Polygonaceae*	*Rumex crispus*	petiole	*Rumex hydrolapathum*	petiole

In the shaded-open habitat contrast, species were carefully selected as shaded or open-habitat species if they predominantly occur in habitat with or without a tree canopy, respectively. A similar selection criterion for was used for the dry-moist habitat contrast, where by the distinction was made between well-drained vs. continuously moist habitats. The selection procedure was informed by field experience, regional floras and Ellenberg ecological indicator values [22], [23]. See Table S1 for the respective Ellenberg values for light and moisture for the species used in the experiment.

The experiment was performed with seeds from previous collections (2004–2005) and some additional species were purchased from commercial seed suppliers. During field collection, freshly matured seeds were collected from various locations in the vicinity of Lund, southernmost Sweden, i.e. all sites having almost identical climatic conditions and similar soil type. Seeds were obtained from several plants of a single population per species. Collected seeds were air-dried at room temperature and stored in paper bags at room temperature until further use.

### Experimental design and conditions

The experiment was performed during May and June 2006 in a greenhouse, where temperatures gradually increased from 25 to 35°C (daytime) and 13 to 16°C (nighttime) during the experiment. Photosynthetically active radiation (PAR) at midday outside the greenhouse varied from about 170 µmol m^−2^ s^−1^ on an overcast day, to about 1450 µmol m^−2^ s^−1^ on a cloudless, sunny day. The ambient light climate in the greenhouse was less variable due to the automatic blinds which avoided excess radiation and was about 150–250 µmol m^−2^ s^−1^ at midday, depending on the weather conditions, and the red to far red ratio (R:FR) was 1.15, which is the typical ambient value [24].

The seedling experiment was performed in a fully factorial design with a watering treatment (low and high frequency of watering) and a light treatment (low, intermediate and high). Seedlings were placed on two adjacent elongated benches with one watering treatment each. The levels of the light treatment were replicated on each greenhouse bench. To create the light treatments, seedlings were placed under frames covered with different plastic films, approximately 40 cm above the benches. The high light treatment had a colourless transparent plastic film, which reduced ambient greenhouse PAR to 75% and did not affect the R:FR ratio. In the intermediate light treatment, frames were covered with green plastic film (#138 Lee Filters, Andover, UK) which reduced ambient greenhouse PAR to 56% and the R:FR ratio to 0.65. The low light treatment was applied using another green plastic film (#122 Lee Filters, Andover, UK), reducing ambient greenhouse PAR to 30% and the R:FR ratio to 0.21. The light extinction rates in the three treatments are not easily compared to light conditions in forests, but would correspond to approximately 70 %, 40% and 10% of open conditions on an overcast day, but 13%, 7% and 2% on a cloudless, sunny day. The values for light extinction under dense forest canopies given in the literature vary between less than 10% down to less than 3% [25], [26].

Seeds of species known to need a period of chilling to relax seed dormancy [9] were subjected to a cold stratification treatment for 11 weeks. In May 2006, seeds were germinated in Petri dishes on moist filter paper. Five seedlings in the cotyledon stage were transplanted within 2 days after germination into 9 cm pots filled with a nutrient enriched peat soil, equally spaced from each other and the sides of the pot. Seedlings of all species were transplanted into the pots within three days of each other (*Geum rivale* one week later because of slightly delayed seed germination). Five replicate pots per species per treatment combination were used. The pots were randomly placed beneath their respective light treatments with sufficient distance among pots to prevent interaction between individuals from different pots. The pots were regularly relocated. The pots were watered every second day (high frequency) or every 6–9 days (low frequency). At each watering event, the bench was filled with one cm water and pots were allowed to absorb water through their drainage holes for 30 minutes after which excess water was drained off from the bench.

After 34 days, seedling mortality was recorded and the surviving seedlings were harvested. On all individuals, total height was measured as well as longest internode, the longest petiole or the longest leaf sheath depending on the morphology of species (Table 1). A representative sample of 2–5 fully expanded leaves per pot from different individuals was collected and scanned on a flatbed scanner. Plant material was dried at 40°C until constant weight. Seedling dry weight was determined and the SLA was calculated from the dry weight and the surface area of the sampled leaves following the standard procedure [27]. SLA was averaged for each pot. Height measurements are unfortunately missing for *Rumex* species and SLA values are missing for both *Achillea* and *Rumex* species. Biomass at harvest is used as an estimate of growth rate, since all plants started as newly germinated seedlings in the cotyledon stage.

### Data analysis

The nature of the watering treatment (each level was bound to a greenhouse bench) together with space and resource limitation due to the large number of species and replicate seedlings were constraining the statistical analysis. The levels of the watering treatment were not replicated in space. However, the spatial configuration of two adjacent elongated benches, combined with the uniform light and temperature conditions in the greenhouse did not allow for variation between the benches other than the large difference between the watering regimes which we imposed on the seedlings.

Factorial analysis of variance (ANOVA) was used to test for the effects of watering, light and habitat type on height, biomass, SLA, relative elongation measure (internode, petiole, or sheath length) and mortality. Relative elongation for each treatment combination for each species was calculated as the respective internode, petiole or sheath length (see Table 1) standardized to (divided by) the mean value in the high light/frequent watering treatment. Analyses were performed on the shaded-open habitat contrast and the dry-moist habitat contrast separately, as well as for each habitat-group separately. Genus was treated as a random factor in all analyses. In the analysis of mortality and SLA, each pot was considered a replicate, whereas in the other analyses the five seedlings were treated as replicates within pot, which was then used as a random factor nested within each combination of the other factors. SLA, biomass, and height values were log-transformed in order to get normal-distributed data. In addition, the log-transformation assured phylogenetic independence, because it removed the correlations between the pair mean and the pair difference which otherwise would have resulted in non-independence between the congeneric species pairs [28]. Post-hoc Tukey tests were used to identify significant differences between means.

## Results

### Growth (biomass)

**Figure 1:**
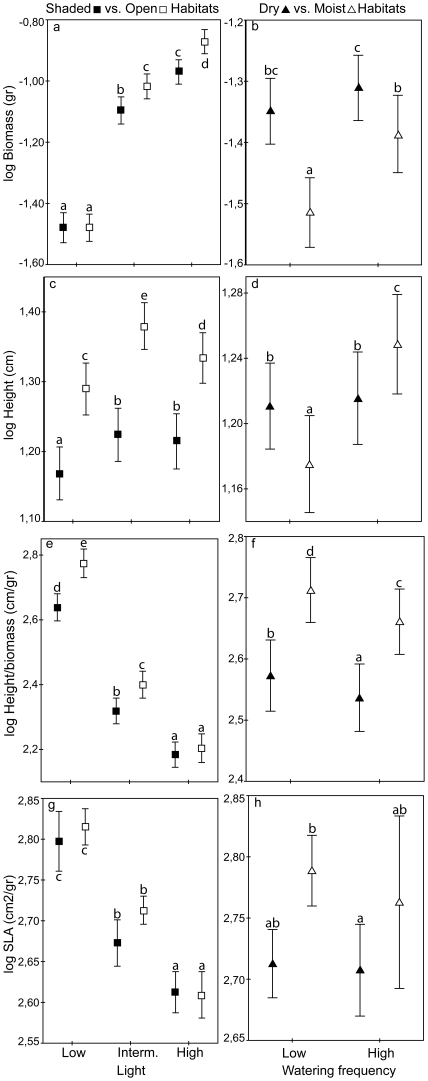
Effect of light and watering treatments on plant traits. Log-transformed values of growth (biomass), height, slenderness (height/biomass) and SLA of species from shaded vs. open habitats (left) and dry vs. moist habitats. Error bars show 95% confidence intervals. Response variables in the open-shaded habitat contrast are plotted against the light treatment and for the dry-moist habitat contrast against the watering frequency. Different letters indicate significant different differences between treatment-habitat combinations (p<0.05).

**Figure 2:**
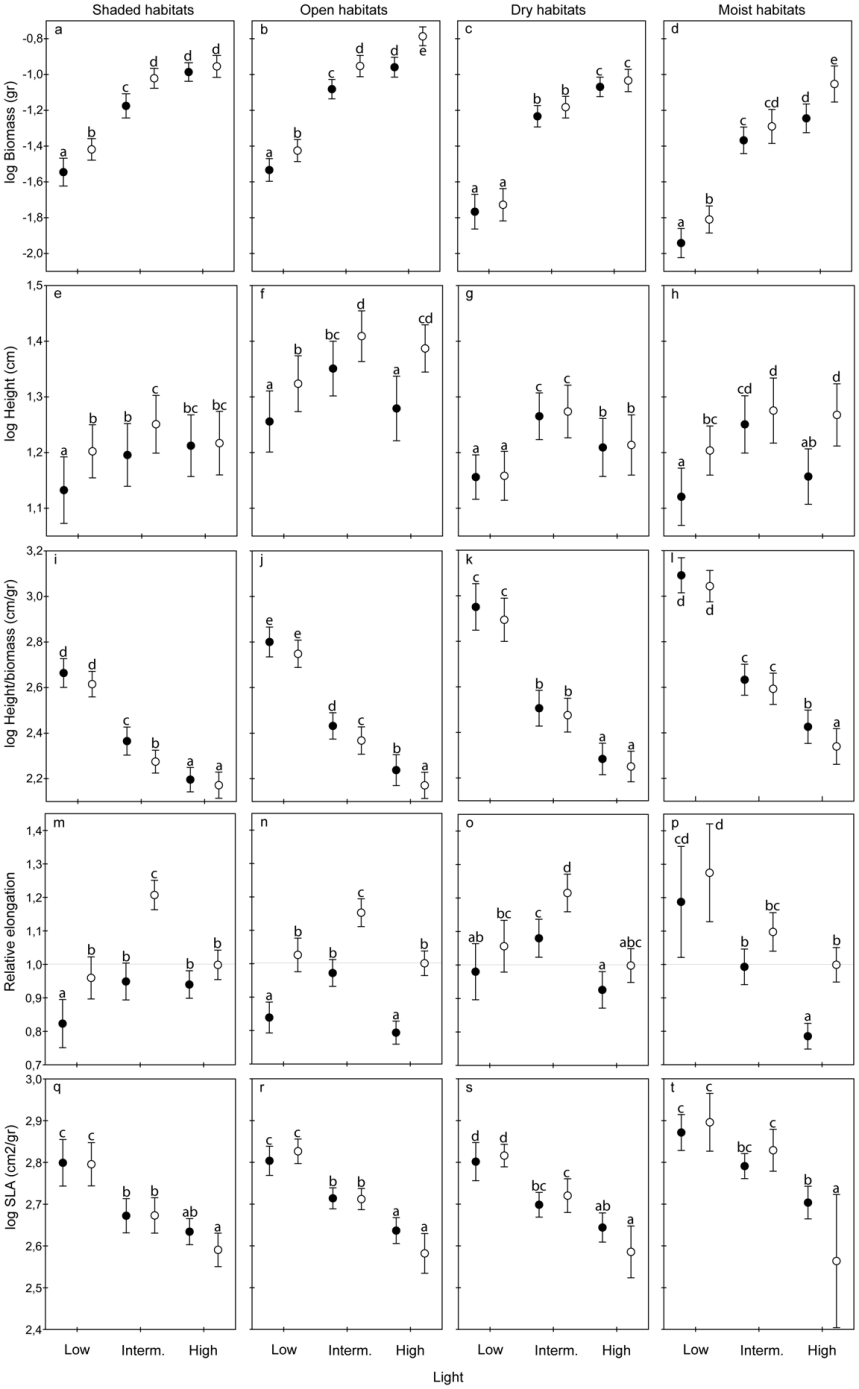
Combined effects of light and watering on plant traits. The combined effects of light and watering frequency (filled circles: low frequency; open circles: high frequency) on growth (biomass), height, slenderness (height/biomass) and SLA response of species from shaded, open, dry and moist habitats. Error bars show 95% confidence intervals. All variables except relative elongation are log-transformed. Different letters indicate significant different differences between treatment combinations (p<0.05). Note that relative elongation is a ratio (see main text more an explanation), so the x-axis of each graph for relative elongation is independent of the x-axis of the other graphs.

Growth was strongly reduced with decreasing light (Table 2, upper panel). Species from open habitats performed better than shaded-habitat species at intermediate and high light (Fig 1a). Dry-habitat species performed better than moist-adapted species across all light levels. Growth was generally reduced with reduced watering frequency (Table 2, upper panel) but not much for the dry-habitat species (Fig 1b). Species from dry habitats had a higher growth rate than those from moist habitats. Open-habitat species performed better than shade-adapted species at both watering frequencies. There was no significant interaction between watering frequency and light on growth in any of the habitats. Reduced watering frequency however, significantly reduced growth in all but the dry-habitat species (Table 2, lower panel, Fig 2a–d).

**Table 2:** ANOVA output table.

Habitat contrast		Biomass		Height		Slenderness		SLA	
		MS	d.f.	MS	d.f.	MS	d.f.	MS	d.f.
Shade/	Habitat	**1.119**	**1**	**5.472**	**1**	**2.699**	**1**	0.024	1
Open	Water	**3.808**	**1**	**1.299**	**1**	**0.952**	**1**	0.013	1
	Light	**30.762**	**2**	**0.685**	**2**	**32.609**	**2**	**0.948**	**2**
	Pot	**0.126**	**343**	**0.035**	**284**	**0.080**	**284**	-	-
	Genus	**11.540**	**5**	**22.186**	**4**	**21.735**	**4**	**0.093**	**4**
	H×W	0.010	1	0.047	1	0.010	1	<0.001	1
	H×L	0.117	2	0.030	2	**0.514**	**2**	0.012	2
	W×L	0.145	2	0.010	2	0.019	2	**0.024**	**2**
	H×W×L	0.179	2	0.081	2	0.024	2	0.002	2
	Error MS	0.056	1095	0.012	1095	0.030	1095	0.008	282
Dry/	Habitat	**1.078**	**1**	0.014	1	**3.993**	**1**	**0.190**	**1**
Moist	Water	**1.507**	**1**	**0.444**	**1**	**0.389**	**1**	0.012	1
	Light	**34.274**	**2**	**1.032**	**2**	**42.887**	**2**	**0.742**	**2**
	Pot	**0.178**	**284**	**0.048**	**225**	**0.091**	**225**	-	-
	Genus	**21.126**	**4**	**14.065**	**3**	**36.936**	**3**	**0.118**	**2**
	H×W	0.461	1	0.215	1	0.013	1	0.004	1
	H×L	0.012	2	0.015	2	0.007	2	0.026	2
	W×L	0.021	2	0.015	2	0.055	2	**0.077**	**2**
	H×W×L	0.046	2	0.063	2	0.026	2	0.011	2
	Error MS	0.050	875	0.012	875	0.024	875	0.012	165

Habitat		Biomass		Height		Slenderness		SLA		Elongation	
		MS	d.f.	MS	d.f.	MS	d.f.	MS	d.f.	MS	d.f.
Shaded	Water	**2.072**	**1**	**0.044**	**2**	**0.487**	**1**	**0.009**	**1**	**4.803**	**1**
	Light	**13.416**	**2**	**0.257**	**2**	**12.214**	**2**	**0.445**	**2**	**2.494**	**2**
	Pot	**0.106**	**169**	**0.031**	**140**	**0.048**	**140**	-	-	**0.186**	**169**
	Genus	**5.871**	**5**	**11.850**	**4**	**9.442**	**4**	**0.306**	**4**	**5.702**	**5**
	W×L	0.189	2	0.044	2	0.035	2	0.007	2	**0.572**	**2**
	Error MS	0.070	534	0.012	534	0.034	534	0.030	140	0.039	653
Open	Water	**1.740**	**1**	**0.942**	**1**	**0.466**	**1**	0.006	1	**7.455**	**1**
	Light	**17.508**	**2**	**0.463**	**2**	**21.097**	**2**	**0.520**	**2**	**2.230**	**2**
	Pot	**0.065**	**169**	**0.022**	**140**	**0.043**	**140**	**-**	**-**	**0.122**	**169**
	Genus	**8.519**	**5**	**10.854**	**4**	**14.880**	**4**	**0.069**	**4**	**1.451**	**5**
	W×L	0.134	2	0.047	2	0.007	2	**0.018**	**2**	0.024	2
	Error MS	0.044	561	0.013	561	0.026	561	0.050	138	0.040	679
Dry	Water	0.149	1	0.020	1	0.073	1	0.002	1	**1.174**	**1**
	Light	**16.576**	**2**	**0.569**	**2**	**20.134**	**2**	**0.282**	**2**	**2.282**	**2**
	Pot	**0.172**	**140**	**0.042**	**111**	**0.078**	**111**	-	-	**0.228**	**140**
	Genus	**7.013**	**4**	**6.309**	**3**	**21.249**	**3**	**0.051**	**2**	**6.133**	**4**
	W×L	0.043	2	0.002	2	0.024	2	**0.014**	**2**	0.068	2
	Error MS	0.047	428	0.008	428	0.025	428	0.040	81	0.051	546
Moist	Water	**1.840**	**1**	**0.656**	**1**	**0.379**	**1**	0.015	1	**3.067**	**1**
	Light	**17.730**	**2**	**0.473**	**2**	**22.865**	**2**	**0.495**	**2**	**6.524**	**2**
	Pot	**0.074**	**140**	**0.033**	**111**	**0.044**	**111**	-	-	**0.708**	**140**
	Genus	**18.203**	**4**	**8.468**	**3**	**18.108**	**3**	**0.384**	**2**	**16.837**	**4**
	W×L	0.023	2	0.100	2	0.015	2	**0.074**	**2**	0.267	2
	Error MS	0.052	447	0.015	447	0.024	447	0.011	82	0.080	563

Results of ANOVAs for growth (biomass), height, slenderness (height corrected for biomass), SLA and relative elongation. All variables except relative elongation are log transformed prior to analysis. The upper panel shows the analysis for the habitat-treatment analysis for both habitat contrasts. The lower panel shows the analysis where the treatment interaction is investigated per habitat separately. Mean squares (MS) and degrees of freedom (d.f.) are reported. Because of the unbalanced design due to seedling mortality, F-ratios for each independent variable were obtained using computed error terms (MS and d.f.) using Satterthwaite's method. Variables with values in bold are significant (p<0.05).

### Plant height and shade avoidance

Plant height varied with light (Table 2, upper panel) and mean height was largest at intermediate light. Open-habitat species grew taller than shade-habitat species (Fig 1c), but no difference in height between species from moist and dry habitats was found. Height was not affected by watering frequency in dry-habitat species, but did increase with increasing watering frequency for moist-habitat species (Fig 1d) as well as the open- and shaded-habitat species. The interaction between habitat (dry-moist) and watering frequency was significant (Table 2, upper panel). Low frequency watering generally reduced plant height, except for the dry-habitat species. The interaction between watering frequency and light was not significant (Table 2, lower panel, Fig 2e–h).

Slenderness (height corrected for biomass) strongly increased with decreasing light (Table 2, upper panel). The interaction between slenderness and habitat (open-shaded) was significant; at high light, slenderness did not differ between seedlings from the open and shaded habitats, but at intermediate and low light, the shaded-habitat seedlings were less slender than the open-habitat seedlings (Fig. 1e). Moist-habitat seedlings were more slender than dry-habitat seedlings across all light levels. Reduced watering frequency increased slenderness in both habitat comparisons (Table 2, upper panel, Fig 1f). Infrequent watering led to an increase in slenderness but not at all light levels and not for the dry-habitat species (Table 2; lower panel, Fig 2i–l). The interaction between watering frequency and light was not significant.

Because of the way relative elongation was calculated (see methods), only analyses for the species within each habitat separately could be performed. Relative elongation was significantly affected by both light and watering frequency (Table 2, lower panel). Reduced watering frequency generally reduced the ability of seedlings to respond to shade (Fig 2 m–p). The intermediate light level elicited the largest response in elongation, analogue to the plant height response, except for the moist-habitat seedlings (Fig 2m–p).

### Specific Leaf Area

SLA increased with decreasing light (Table 2, upper panel). Species from open and shaded habitats did not differ in SLA across the light levels (Fig 1g). Species from moist habitats had a higher SLA than dry-habitat species over all light levels. Watering regime did not affect SLA in any of the habitat comparisons (Table 2, upper panel, Fig 1h). In all habitats, SLA was lowest at combined high light and frequent watering. The interaction between light and watering regime was significant in all habitats except for the shaded habitats (Table 2, lower panel, Fig q–t).

### Seedling mortality

Seedling mortality varied between treatments and habitats (Table 3). In both habitat contrasts, mortality tended to be higher with decreasing light and decreasing watering frequency, but these differences were not significant (statistics not shown). Species from shaded habitats showed significantly higher mortality than species from open habitats (*F*_1,343_ = 5.97, *p* = 0.015), but no difference was found between species from dry and moist habitats (*F*_1,343_ = .2.85, *p* = 0.92).

**Table 3:** Mean seedling mortality values in percentages per habitat and treatment.

Light	Watering	Habitat			
		Shaded	Open	Dry	Moist
High	infrequent	6.0	4.0	6.4	2.4
	frequent	6.0	1.3	4.0	5.6
Intermediate	infrequent	10.0	4.0	4.8	8.8
	frequent	6.0	5.3	8.8	4.8
Low	infrequent	10.0	6.0	12.0	5.6
	frequent	6.0	6.7	7.2	3.2

## Discussion

### Effects of watering regime and shading on plant traits

Are different plant strategies in the seedling phase underlying habitat specialization?

The increase in growth rate with increasing light indicates that biomass production was limited by shade for all species. Species from shaded habitats grew slower than species from open habitats, and also showed a more modest response to increased irradiation. This confirms the idea that shade-tolerant seedlings are adapted to conserve energy by growing slowly in order to secure long-term survival. This is in contrast to a strategy to maximize growth, which is the more successful strategy for species from less shade-stressed environments [29], [30]. Shade-tolerant plants are adapted to efficiently harvest light under constant low irradiance by increasing net carbon fixation per unit leaf protein [31]. This ability is, among other things, provided by thin leaves, which have a low internal self-shading, and a low light compensation point [32], [33], [34].

Open habitat species were relatively more slender at higher light than their shade-adapted congeners and already exhibited a shade-avoidance response at intermediate light. This strategy of elongation increases plant performance only when the investment in vertical growth leads to increased light interception. Plants from open habitats perceive shade from neighbouring herbaceous vegetation, and may improve their light climate greatly by growing taller and catching up with or overtopping their neighbours. Shade-avoidance, however, was also manifest in the species from shaded habitats at deep shade, despite the fact that elongation is generally less adaptive for forest species, since they are shaded by the tree canopy [18], [35], [36].

The costs of expressing shade avoidance are reduced water use efficiency due to a lower root to shoot ratio [37]. Expressing shade avoidance traits could lead to an increased vulnerability to drought stress and the adaptive value of petiole and stem elongation is generally reduced when plants experience drought stress [38]. Reduced watering limited shade-avoidance expression in species from both open and shaded habitats; relative elongation and plant height were lower. Dry-habitat species showed no difference in shade avoidance between low and high watering, except for relative elongation at intermediate light. This is probably due to the minimal fitness advantage of elongation in dry habitats, where increased elongation leads to increased water-loss.

Our hypothesis that shade-adapted species would have a higher SLA than open-habitat species was confirmed by the results, although shade-adapted species are often reported to have thinner leaves and thus a higher SLA [39]. High plasticity in SLA in response to varying light levels of species from both shaded and open habitats was reported by Haberlandt already more than a century ago [40] and is also manifest among our study species, but the contrasting environments have apparently not selected for differences in mean SLA between these two groups.

The results confirm our hypothesis that dry-habitat species are less affected by drought than are moist-habitat species. However, it was contrary to our expectations that dry-habitat species outperformed moist-habitat species in biomass production. We expected that species confined to drought-prone habitats would adopt a more conservative growth strategy analogous to that of shade-adapted species [30]. The lower SLA of the dry-habitat species is an indicator of this conservative growth strategy, as SLA in general is correlated with growth rate [19], [41]. Growth rate, however, is also a function of assimilation rate and dry matter content [19]. The lower SLA of dry-habitat species could thus also be attributed to higher water-use efficiency due to a smaller leaf surface reducing evapotranspiration. Another explanation could be that adaptation to dry sites often involves rapid growth in those periods of high water availability [17], although this is not confirmed by our results.

In our study, SLA was greatly influenced by the light environment. SLA increased with decreasing light availability. An increased leaf area increases evapotranspiration, and a smaller increase in SLA with reducing light would be expected under the low watering regime, but this was not observed in our study.

### Interactive effects of drought and shade on plant fitness

The absence of a significant interaction between the watering treatment and the light treatment on growth, plant height and slenderness in all habitats suggests that the effects of drought do not amplify the negative impact of shade on the species. An amplified effect could be expected since a greater allocation to shoots (reducing root to shoot ratio) and specifically to leaves and leaf area in response to shade or as adaptation to shaded habitats could compromise resistance to drought [12]. The experimental design could have partly counteracted a possible amplifying effect of drought on the effect of shade. Since the watering intervals were equal across irradiances, this design could potentially impose a temporarily stronger drought in high irradiance, as the plants may deplete water more quickly from their pots due to high transpirational load, and, with the plants in high irradiance becoming larger than those in shade, this effect would be aggravated. However, with our experimental setup, it is not possible to disentangle the effects of light itself and the secondary effect of increased desiccation.

Drought, however, reduced growth and height significantly for the shaded-habitats plants at the low and intermediate light levels, but not at high light. This may suggest a trade-off between drought and shade tolerance in species from shaded habitats resulting in amplified effects of drought at low light levels. Among open-habitat species and moist-habitat species, drought and shade appeared to impact growth and orthogonally, corresponding to the findings of [42] that drought has a proportional effect on growth and height independent of the light level. A third variant was observed among the dry-habitat species, where watering frequency had no impact on growth and height across the light levels. This indicates that the dry habitat-species are apparently adapted to grow at low moisture levels and are not able to profit from a higher water availability. Facilitation by shade, which would hypothetically relieve evapotranspiration by reducing temperatures, did vary with the shading treatments although the temperatures beneath the plastic shading films only differed marginally.

Although the watering regime strongly influenced the water availability in the soil, we had no direct control of the actual water availability in the pots as this was also influenced by the plant species and plant biomass in the pot. Pots with larger plants dried out faster than pots with smaller plants. When interpreting the results, it should further be noted that the ‘high’ light level in our treatment is high relative to the other levels, and similar to full day light as perceived by plants in nature on an overcast day. The ambient greenhouse light levels of 150–250 µmol m^−2^ s^−1^ at midday, however, correspond to normal greenhouse light conditions. A higher light intensity at the high light level could possibly have revealed patterns and differences between species from different habitats that now are not shown, like bigger differences in SLA with increasing light and possible more pronounced trade-offs between shade and drought tolerance. Photoinhibition, which can occur when light exceeds the saturation point, did not occur in our study. Under field conditions, especially plants from shaded habitats would be affected by high light as their saturation point is low and the leaves are adapted to function under low light levels.

### Seedling mortality

Seedling mortality was low in our experiment; the only significant difference found was between open and shaded habitats, although mortality also tended to be higher with increasing drought and decreasing light. Moles and Westoby [43] screened the literature and found herbivory, drought and fungal attack to be the major causes of seedling mortality in nature, whereas physical damages and competition with established vegetation and other seedlings were of minor importance. However, in our experiment, it seems likely that a shortage of resources led to competition and subsequent mortality of some seedlings, as the low mortality rates indicate that resource shortage, e.g. drought, was apparently not so severe to kill of a large number of seedlings.

### Species traits and habitat specialization

We conclude that the two focal habitat contrasts in our study have imposed divergence between species in growth related traits. Plants from contrasting habitats are differently affected and constrained by shortages of light and water, which, among other factors, contribute to their habitat specialization. Segregation of plants along gradients of light or water availability, however, is also influenced by other factors such as nutrient availability, competition, disturbance, pathogen pressure and herbivory. Regeneration of plants, like seed germination has been shown to be very important for habitat specialization in forest herbs [8], [9]. This study shows that seedling phase of species contribute to habitat specialization, and suggest that also other phases in the plant life cycle than the established phase are important in adaptive specialization.

## Supporting Information

Table S1 **Ellenberg indicator values for light and moisture for the species used in the experiment.** Ellenberg [22] indicator values for light and moisture for the species used in the experiment. Some species have no indicator value in this system. For convenience, we have added the similar values from Hill's system [23] of Ellenberg-values adapted to British conditions in parentheses. In both systems, the LIGHT indicator value has an ordinal scale from 1–9 (from deep shade to full light) and the MOISTURE indicator value has an ordinal scale from 1–12 (drought indicators to submerged hydrophytes).
(DOC)
