# Supplementary material for: Seedling Stage Strategies as a Means of Habitat Specialization in Herbaceous Plants
Source: PLoS One. 2011 Jul 29;6(7):e23006. doi: 10.1371/journal.pone.0023006 (PMC3146528; doi:10.1371/journal.pone.0023006)
Supplement: Table S1 — Ellenberg indicator values for light and moisture for the species used in the experiment. Ellenberg [22] indicator values for light and moisture for the species used in the experiment. Some species have no indicator value in this system. For convenience, we have added the similar values from Hill's system [23] of Ellenberg-values adapted to British conditions in parentheses. In both systems, the LIGHT indicator value has an ordinal scale from 1–9 (from deep shade to full light) and the MOISTURE indicator value has an ordinal scale from 1–12 (drought indicators to submerged hydrophytes). (DOC) [file pone.0023006.s001.doc]

Table S1. Ellenberg indicator values for light and moisture for the species used in the experiment.

| Family | Open-habitat species | Ellenberg light | Ellenberg moist | Shaded-habitat species | Ellenberg light | Ellenberg moist |
| --- | --- | --- | --- | --- | --- | --- |
|  |  |  |  |  |  |  |
| *Poaceae* | *Bromus hordeaceus* | 7 | (4) | *Bromus benekenii* | 7 | (4) |
| *Cyperaceae* | *Carex ovalis* | 7 | 7 | *Carex sylvatica* | 7 | 7 |
| *Poaceae* | *Festuca arundinacea* | 8 | 7 | *Festuca gigantea* | 8 | 7 |
| [*Rosaceae*](http://luirig.altervista.org/famiglie/rosaceae.htm) | *Geum rivale* | 6 | 8 | *Geum urbanum* | 6 | 8 |
| *Polygonaceae* | *Rumex crispus* | 7 | 7 | *Rumex sanguineus* | 7 | 7 |
| *Caryophyllaceae* | *Silene latifolia* | (7) | (4) | *Silene dioica* | (7) | (4) |
|  |  |  |  |  |  |  |
|  |  |  |  |  |  |  |
|  | Dry-habitat species |  |  | Moist-habitat species |  |  |
|  |  |  |  |  |  |  |
| *Asteraceae* | *Achillea millefolium* | 8 | 4 | *Achillea ptarmica* | 8 | 4 |
| *Poaceae* | *Agrostis capillaris* | 7 | (5) | *Agrostis stolonifera* | 7 | (5) |
| *Cyperaceae* | *Carex ovalis* | 7 | 7 | *Carex lepidocarpa* | 7 | 7 |
| [*Rosaceae*](http://luirig.altervista.org/famiglie/rosaceae.htm) | *Geum urbanum* | 4 | 5 | *Geum rivale* | 4 | 5 |
| *Polygonaceae* | *Rumex crispus* | 7 | 7 | *Rumex hydrolapathum* | 7 | 7 |
